# Treatment of Inappropriate Sexual Behavior in Dementia

**DOI:** 10.1007/s11940-016-0425-2

**Published:** 2016-08-11

**Authors:** Riccardo De Giorgi, Hugh Series

**Affiliations:** 1 Department of Psychiatry, University of Oxford, Warneford Lane, Oxford, OX3 7JX UK; 2 Oxford Health NHS Foundation Trust, Warneford Hospital, Oxford, OX3 7JX UK

**Keywords:** Inappropriate sexual behavior, Dementia, Older adults, Treatment, Systematic review

## Abstract

Inappropriate sexual behavior (ISB) is a relatively common and potentially disruptive form of behavior in people with dementia. It can cause considerable distress and put placements and people at risk. Yet it is poorly researched and understood. In addition to non-pharmacological approaches to management, a wide range of classes of medication has been used in ISB, and the results have been reported as single cases or short series, though none has been the subject of a randomized clinical trial, in part because of the lack of a well-defined method of observing and measuring ISB, as well as the significant ethical considerations. Pharmacological treatments for which there is low-level evidence of efficacy in the literature include antidepressants, antipsychotics, anticonvulsants, cholinesterase inhibitors, hormonal agents, and beta-blockers. None of the drugs discussed here is licensed for use in ISB, and elderly people, particularly those with dementia, are at high risk of adverse effects. Caution is advised before using medication in this group of people. It is important to consider alternative non-pharmacological treatments, as well as discussing issues of ethics and consent with those involved, before initiating treatment. It is helpful to identify and monitor target symptoms. Pharmacological treatments should be started at low dose and titrated up slowly and carefully. Nevertheless, in some situations, medication may provide a useful part of a management plan for ISB.

## Introduction

Sexuality is one of the basic needs in human life. Although sexual activity often decreases in elderly people, sexual interest may not [1]. Despite this, sexual expression among older adults is often perceived as non-existent, humorous, grotesque, or even sordid and disgusting [2]. However, as in younger people, not all sexual behavior in older people is inappropriate.

In elderly patients with dementia, a combination of cognitive deterioration, worsening judgment, and personality changes probably contributes to changes in sexual attitude and behavior. The most common alteration reported in people with dementia is apathy and indifference to sex [3]. Nevertheless, inappropriate sexual behavior (ISB, also known as sexually disinhibited behavior, or hypersexuality) has been consistently described in most dementia syndromes [4].

## Definition

The concept of ISB has developed over time [5••]. Johnson et al. define it as, “a disruptive behavior characterized by a verbal or physical act of an explicit or perceived sexual nature, which is unacceptable within the social context in which it is carried out” [6], while de Medeiros et al. suggest that “ISB is a specific sexual behavior marked by apparent loss of control or intimacy-seeking misplaced in the social context or directed towards the wrong target; behavior may be not sexual in its form but in its suggestion” [7]. ISB should be seen as a part of the symptom cluster of behavioral and psychiatric disturbances associated with dementia, which is disruptive and distressing, and impairs the care of the patient [8].

## Classification

ISB can include sex talk (i.e., using foul language that is not in keeping with the patient’s premorbid personality), sexual acts (i.e., acts of touching, grabbing, exposing, or masturbating, which can occur in private or in public areas), and implied sexual acts (i.e., openly reading pornographic material or requesting unnecessary genital care) [9].

ISB can be divided in conventional and non-paraphilic (i.e., sexual interest arise within socially and culturally accepted boundaries), versus unconventional and paraphilic (i.e., sexual arousal that deviates from previous restrictions, for instance, involving children, animals, and non-consenting people) [10].

In one observational study, sexual behavior was coded as follows: appropriate (e.g., sitting close to someone with arms or legs touching, kissing, stroking someone on the face, hands, or arms), ambiguous (e.g., being undressed outside the bedroom or bathroom, rubbing up against another person, touching self on breasts or genitals in public), and inappropriate (e.g., making explicit sexual comments, touching someone other than partner on breast or genitals, touching partner on breast or genitals in public, exposing breasts or genitals in public) [11]. Despite its thoroughness, this classification has several flaws. Firstly, the concept of appropriateness varies among individuals and can be affected by numerous elements, such as religious beliefs or prevailing societal views of elderly persons [12]. Acts like public undressing or genital touching may be misinterpreted as sexual, when in fact they can result from pain, discomfort, hyperthermia, or attempts to be freed from a restrained environment [6]. Normal needs for sex and intimacy in older adults are frequently regarded as an inappropriate increase in sexual drive [8]. Lastly, inappropriateness often stems from judgmental mind-sets and arbitrations of the observers (clinical staff, family, and other residents), rather than the sexual activities themselves [13].

## Epidemiology

The overall prevalence of behavioral and psychiatric symptoms of dementia is 50–80 % [14]; thus, the majority of people with dementia may experience some of these symptoms. The occurrence of ISB in demented individuals reportedly ranges from 7 to 25 %, with higher prevalence in residents of skilled nursing facilities and in patients with more severe cognitive impairment [9, 15]. Physical manifestations appear to be more frequent in males [16], whereas women seem more verbal [17].

ISB can be a threat to the mental and physical health of patients and others [18]; for example, repeated masturbation can cause genital trauma [19]. These activities can cause anxiety, distress, and embarrassment in caregivers, often disrupting the continuity of care at home and leading to confinement at home or institutionalization [20]. Finally, sexualized conducts may cause a conflict between ethical and legal responsibilities of clinicians, since hindering sexual expression can be seen as a violation of the principle of autonomy [18]; therefore, the capacity to consent needs to be assessed [21].

## Etiology

Many neurobiological and psychosocial factors can contribute to the onset and maintenance of ISB. The neurobiological basis of sexuality is likely founded on complex interactions between different brain systems, neuroendocrine factors, and neurotransmitters.

Four brain systems have been implicated in the neurobiology of ISB: the frontal lobes, the temporo-limbic network, the cortico-striatal circuits, and the hypothalamus [8]. Dysfunction of the frontal lobes, often seen in dementia, is known to trigger disinhibited behaviors [22], and it has been associated with ISB [23]. Bilateral lesions of the temporal regions result in Klüver-Bucy syndrome, which includes autoerotic behavior and hyperorality [24]; hypersexuality has been reported after temporo-limbic strokes [25], tumors [26], and epilepsy [27]. Some authors have conceptualized hypersexuality as being obsessive-compulsive in nature, thus involving cortico-striatal circuits, as in Huntington’s disease [28], Tourette’s syndrome [29], and Wilson’s disease [30]. Lastly, lesions to the right hypothalamus and periventricular area can cause manic symptoms, including increased sexual drive [31].

Neuroendocrine factors affecting ISB include androgens, estrogens, progesterone, prolactin, oxytocin, cortisol, pheromones, and neurotransmitters and neuropeptides including nitric oxide, serotonin, dopamine, adrenaline, noradrenaline, opioids, acetylcholine, histamine, and gamma-aminobutyric acid [32].

Psychotropic drugs such as levodopa, benzodiazepines, and alcohol have all been linked with increased agitation and sexual disinhibition [33].

Psychosocial issues often contribute to the onset of ISB in predisposed demented patients; these factors include mood instability, premorbid patterns of sexual activity and interest, lack of habitual sexual partner or misidentification of someone else as usual partner, lack of privacy, understimulating or unfamiliar environment, misinterpretation of cues, and potentially many others [33]. These aspects are explored more fully by Benbow and Beeston [34••].

## Assessment

A good management plan should be based on a thorough assessment of a patient’s personal, clinical, and sexual history, including who is involved, what form the ISB takes, when and where it occurs, how frequent it is, and other factors such as potential precipitants and consequences of the behavior [33]. If the patient is severely impaired, then a history should be obtained from the caregivers or family members [8]. Lawrie and Jillings emphasize the importance of considering the appropriateness or inappropriateness of a sexual behavior according to the larger context, and managing contributory factors such as boredom, loneliness, or misinterpreted gestures of communication [35].

Some behavior measurement tools for patients with dementia include items relating to ISB. The Ryden Aggression Scale contains a section on sexually aggressive behavior including making obscene gestures, touching body parts of another person, hugging, intercourse, or kissing [36]. The St Andrew’s Sexual Behavior Assessment (SASBA) is based on continuous direct observation of four categories of ISB, each with four levels of severity, helping clinicians to standardize their documentation of ISB [37].

A systematic physical and mental state examination and a review of the current medication regime must always be carried out [5••]. In the case of an abrupt onset of the ISB, laboratory testing and neuroimaging studies to evaluate for delirium or a new structural lesion should be considered [8].

## Aims

This study systematically reviews the published literature on the current treatment options for ISB in older adults with dementia in order to support the clinician in managing ISB.

## Methods

A systematic research of the databases MEDLINE, Google Scholar, and Cochrane Library was conducted for publications on ISB in dementia until April 2016. Using the MeSH terms “inappropriate sexual behavior,” “dementia,” “Alzheimer’s disease,” “frontotemporal dementia,” “vascular dementia,” “Lewy body disease,” and “mild cognitive impairment,” we retrieved 162 records, to which other 18 and 24 articles were added using respectively the non-MeSH terms “sexual disinhibition” and “hypersexuality.” We performed a hand-search of the reference list of the studies identified, especially previous reviews, which identified a further nine significant publications. We eliminated 24 duplicate records. We looked for original articles, namely case reports, case series, case-control and cohort studies, and randomized controlled trials; however, we also considered previous reviews and expert opinions published where evidence-based studies were lacking. Finally, we selected 37 original articles which contained information regarding treatment of ISB in dementia. We present the evidence divided into non-pharmacological and pharmacological treatments.

## Results

### Non-pharmacological treatment

Although there are few reports of non-pharmacological interventions and there is a lack of evidence for their efficacy, there is a general agreement that they should be the first-line treatment for ISB in elderly people with dementia [20], principally because of concerns about safety and ethics [38]. However, clinicians often manage problematic behavior with medication because of the ease of administration, perceived efficacy [39], and lack of trained staff members [40].

Non-pharmacological treatment can be divided into environmental, behavioral (or cognitive-behavioral), and educational. These interventions should involve not only the patients but also families and the nursing staff for institutionalized subjects, bearing in mind that the aim is to try and promote an appropriate manifestation of sexual behavior rather than an eradication of it [41•].

#### Environmental approaches

Despite a lack of studies, making changes in the environment, with adjustment of misinterpreted social cues, is often regarded as a simple and effective intervention; for example, switching from a female to a male staff member, or avoiding overstimulating television or radio programs or magazines can be helpful [41•]. Likewise, in nursing homes, single rooms and provision for conjugal visits may reduce the frequency of ISB by satisfying the patient’s normal sexual drive [41•].

#### Behavioral approaches

Few studies of behavioral approaches have been published. A case series of ten people with dementia and ten age- and sex-matched controls showed that consistent redirection and enhanced communication through an interpreter was associated with a reduction of ISB [23]. Other authors have suggested that sensitive explanation to the patient on why such behavior is unacceptable, as well as verbal or physical redirection, is beneficial. In institutions, when a resident enters the bed of another resident, simply redirecting the person to their room may be sufficient, since this conduct could arise from a search for intimacy without sexual purposes, or disorientation [41•].

A variety of distraction techniques are recommended for controlling ISB: in the context of understimulating settings, provision of social activity (snacks, drinks, conversation, exercising, and walking) can reduce boredom [41•]; similarly, involvement in crafts to occupy the hands can prevent inappropriate touching or public masturbation [42]. A case report of a 68-year-old man with dementia in residential care employed a different stratagem: when this man became agitated and inappropriately touched staff and female residents, he was isolated from female residents and unsuccessfully trialed on different antipsychotics; then, he was given a stuffed toy replica of the Pink Panther, and he started to fondle this puppet inappropriately, becoming less intrusive with people [43].

Referral to specialized psychogeriatric services may be useful, although not always available. A controlled case series comprising 33 patients with behavioral and psychotic symptoms of dementia (including, but not restricted to, ISB) and 22 matched controls compared usual care to individualized assessment and care delivered by a multidisciplinary team, such as a psychiatric nurse with access to older adults psychiatry and geriatric medicine specialists. Access to the multidisciplinary team was effective and associated with less use of psychotropic medications [44].

Cognitive-behavioral therapy (CBT) for ISB can include re-education of the patient about social norms, encouragement to explore the intentions behind each behavior in order to address cognitive distortions, and negative conditioning techniques; however, this approach can be challenging in people with significant cognitive impairment [45], and there is a lack of published data.

In patients with a tendency for exhibitionism or public masturbation, practical solutions such as clothing that opens at the back or lacks zippers have been proposed [18]. Such techniques are controversial because of their ethical implications [46].

#### Psycho-education (family, caregivers, staff)

Evidence from three studies shows that education of family, caregivers, and staff about sexuality and aging is associated with more permissive attitudes and benefit for people with ISB [47–49]; more specifically, sex-education programs, which emphasize the need for normal sexual expression while preventing inappropriate conducts, seem to add to the quality of life of a person with dementia.

The partner, who often needs reassurance that ISB originates from illness and is not a reflection of their relationship, may benefit from supportive psychotherapy, which can help to reframe their partner’s sexual requests as calls for intimacy and reassurance [50].

### Pharmacological treatment

Despite pharmacological treatments being more frequently used than non-pharmacological approaches [51], there are no randomized controlled trials for any of the drugs commonly prescribed, with all current evidence emerging from case series and case reports.

Pharmacological treatments for which there is evidence of efficacy in literature include antidepressants, antipsychotics, anticonvulsants, cholinesterase inhibitors, hormonal agents, and beta-blockers.

In older patients, an important rules of prescribing is “start low, go slow,” monitoring carefully for side effects and response. Where possible, it is helpful to identify target symptoms or behaviors which can be quantified, in order to assess response. Many older people are prescribed many agents simultaneously, and so the possibility of drug interactions must be considered in each case. None of the agents discussed here is licensed for use in ISB, and so the physician must carefully consider their use and advise patients and caregivers of risks and benefits.

#### Anxiolytic agents

Tiller et al. report an 80-year-old person with vascular dementia who was treated with buspirone 5 mg three times daily: improvement of ISB was reported after only 5 days; this treatment was continued for 6 weeks, and no relapse was observed within 1 month from stopping [52].

We have not identified any reports of benefits of benzodiazepines in elderly people with ISB. Caution is required as these agents may cause paradoxical reactions with increased agitation in the elderly [53], and they may worsen cognitive impairment and cause dependence.

#### Antidepressants

The concept of using antidepressants for the treatment of ISB derives from their known anti-libidinal effects [54], as well as their anti-obsessional properties [55].

Selective serotonin reuptake inhibitors (SSRI) are commonly used as first-line agents due to their superior safety and tolerability profile in the elderly [56]. Reported results are inconsistent. Paroxetine 20 mg was used with beneficial effect within 1 week and no substantial side effects in a 69-year-old man with ISB and alcoholic dementia, who had not responded to haloperidol, chlorpromazine, lorazepam, lithium, and nortriptyline [57]. A more recent case report of a 90-year-old woman residing in a nursing home describes treatment failure with paroxetine, whereas a sharp reduction of aggressive and sexually disinhibited behavior was seen with citalopram 20 mg [58]. A similar positive outcome was observed in 85-year-old man with dementia treated with citalopram at the same dose [59]. A 76-year-old person with vascular dementia treated with citalopram did not respond, whereas mirtazapine up to 30 mg was effective [23].

Among tricyclic antidepressants (TCA), only the use of clomipramine has been reported in ISB. In one case report, two elderly people with dementia and ISB responded to clomipramine 150–175 mg after no response to thioridazine; however, one of these patients had been initially prescribed 200 mg of clomipramine which had to be stopped and re-titrated due to postural hypotension [60].

In four males with dementia who had failed to respond to several first-generation antipsychotics and benzodiazepines, a good response was reported for all subjects with trazodone between 100 and 500 mg [61].

The most common side effects associated with the use of SSRIs are gastro-intestinal problems, headache, and hypersensitivity reactions. There is a low but definite risk of serotonin syndrome if prescribed in combination with other serotonergic agents. If discontinued abruptly, some patients experience a withdrawal syndrome.

Tricyclic antidepressants can have troublesome side effects in older people. There is a risk of postural hypotension, which can lead to falls, and anticholinergic effects including constipation, dry mouth, and urinary retention. They can have serious cardiac effects and can be fatal in overdose.

Costs of antidepressants are relatively low, although can be higher for some newer agents.

#### Antipsychotics

Antipsychotics are commonly used for treating behavioral and psychotic symptoms of dementia (BPSD), although this indication is not licensed and the US Food and Drug Administration has issued two black box warnings about the use of respectively second-generation [62] and first-generation antipsychotics [63] in elderly demented people.

There are two case reports of the use of quetiapine for treating ISB. One was an 85-year-old man with dementia, parkinsonism, and compulsory masturbation to the point of self-harm; he showed no response to hormonal treatment and paroxetine, but symptoms disappeared after using quetiapine 25 mg after just 2 days, and did not recur in a follow-up period of 2 months, with no side effects reported, nor worsening parkinsonism [64]. The second was a 61-year-old woman with Lewy body dementia, who showed a reduction of ISB after treatment with quetiapine titrated up to 75 mg [65].

Two case reports describe the successful use of haloperidol. A 70-year-old man who presented with excessive masturbation following a frontal lobe injury showed a marked reduction of symptoms with haloperidol 3 mg [66]. A 90-year-old man with Alzheimer’s disease and an array of behavioral symptoms including urethral masturbation with foreign bodies and sexual disinhibition had a good response to haloperidol 1.5 mg [67].

Side effects of antipsychotics can be very significant. There is a small but definite increased risk of stroke or cardiac events in people with dementia, and this has led to national advice in many countries to avoid their use in dementia wherever possible. Sedation and impairment of cognitive function is common. Atypical antipsychotics can produce weight gain and a metabolic syndrome. Older agents may have anticholinergic effects and more rarely cause dermatological, hematological, or endocrine problems.

Older antipsychotic agents are mostly low in cost, but newer ones may be substantially more expensive.

#### Anticonvulsants

Anticonvulsants have been trialed for the treatment of ISB in dementia; however, the underlying mechanism by which they affect sexual function is still poorly understood [68].

Gabapentin is known to cause reduced libido, anorgasmia, and erectile dysfunction [42]. Three case reports describe reduced ISB in people with dementia treated with gabapentin [69–71].

Carbamazepine has been associated with lower testosterone levels in young women with epilepsy [72]. There are two case reports of its use in ISB. A 78-year-old man with Alzheimer’s disease was unsuccessfully treated with donepezil, galantamine, and pipamperone, while carbamazepine 200 mg proved effective [73]. A 78-year-old man diagnosed with frontotemporal dementia failed to respond to paroxetine. Carbamazepine was started and titrated to a maximum dosage of 800 mg (serum concentration = 6.0 mg/L); the ISB completely abated and did not recur over the subsequent 6 months [74].

Valproate salts have not proved useful in controlling agitation in dementia [75], and currently, there is no evidence that they might work in ISB.

Anticonvulsants can have a wide range of side effects including sedation, gastro-intestinal problems, risk of falls, hypo- or hypertension, and endocrine changes. Gabapentin and its close relative pregabalin are relatively expensive, valproate salts intermediate in cost (depending on formulation), and carbamazepine relatively inexpensive.

#### Cholinesterase inhibitors

Cholinesterase inhibitors may affect sexual function in many ways, including altering testosterone levels, but the exact processes involved are still uncertain [76].

Studies evaluating the effects of cholinesterase inhibitors on ISB are conflicting. Two case reports described a reduction of ISB with rivastigmine treatment up to 3 mg [77, 78], although other reports describe the emergence of ISB in patients taking donepezil [79, 80].

The commonest side effects are gastro-intestinal, but there can be effects on sleep and arousal. Donepezil is available as a generic form which is less expensive than others.

#### Hormonal agents

The use of hormonal treatment for ISB is in the elderly is controversial, especially in terms of social stigma associated with drugs seen as a means of “chemical castration” [8].

##### Antiandrogens and other drugs with antiandrogenic activity

Antiandrogens may reduce androgen levels or inhibit binding at androgen receptors.

Medroxyprogesterone (MPA) does not bind to androgen receptors, but indirectly decreases the level of testosterone by inhibiting the secretion of pituitary luteinizing hormone (LH) and follicle-stimulating hormone (FSH). One case report describes four patients aged between 75 and 84 whose ISB did not respond to first-generation antipsychotics, but stopped 1 week after administration of MPA 300 mg intramuscularly on a weekly basis for 1 year, with a 90 % reduction in serum testosterone levels, which returned to the normal range on stopping MPA [81]. One year later, the same author published another case report of four patients, this time treated with oral MPA 100 mg, obtaining similar results and a complete long-term remission of ISB in three out of four patients. One subject relapsed, but symptoms were much milder than before [82]. Another case report for two patients, aged 72 and 84, who failed to show improvement with thioridazine, describes remission with MPA 100–200 mg intramuscularly fortnightly [83]. Similar results are reported in an 86-year-old man with Alzheimer’s disease and ISB [84]. A man aged 76 with alcoholic dementia and ISB was initially responsive to paroxetine, but the response faded. Serial treatments with MPA up to 750 mg weekly then restored a response: lower doses failed to reduce testosterone levels [85]. A case series describes five people who were all successfully treated with MPA 100–300 mg intramuscularly fortnightly with no side effects reported [86]. Bardell et al. report successful treatment with MPA in three elderly patients with ISB [23].

Cyproterone acetate (CPA) inhibits the interaction between endogenous androgens and androgen receptors, as well as reducing the biosynthesis of androgens. Effective treatment of ISB with CPA 50 mg was reported in a 60-year-old man with AIDS-related dementia [87]; however, another case report described unsuccessful CPA treatment in a 70-year-old with dementia, with additional numerous adverse effects impacting on mobility [88]. CPA 10 mg was used with beneficial effect in two males with dementia-related ISB that had not responded to treatment with antipsychotic or sedative medication. The behavior re-emerged in both people when attempts were made to reduce the dose [89].

Finasteride is a 5α-reductase inhibitor which inhibits the conversion of testosterone to active dihydrotestosterone. In a case series of 11 elderly men with ISB and vascular dementia, it was successful in six subjects within 8 weeks, while the other five patients required alternative treatments, such as propranolol and quetiapine [90].

Cimetidine (H_2_ antagonist), spironolactone (potassium-sparing diuretic), and ketoconazole (antifungal) all show antiandrogenic effects; they have been used in a case series of 17 men and 3 women with ISB. Of these, 14 responded to either cimetidine 600–1600 mg, spironolactone 75 mg, or ketoconazole, or to all three drugs combined together [91].

##### Estrogens

Estrogens inhibit the secretion of LH and FSH, thus diminishing testosterone production.

In one case report of the use of diethylstilbestrol (DES) in a 94-year-old man who presented with very significant episodes of sexual aggression towards other in-patients, there was a response to DES 1 mg within 3 weeks [92].

A large case series reports improvement of ISB in 38 out of 39 people with dementia who were treated with oral estrogen 0.625 mg or transdermal estrogen patches 0.5–0.10 mg [93]. A case is reported of a 75-year-old patient with dementia who failed to respond to thioridazine, but responded to transdermal estrogen within 1 week, which allowed tapering of the antipsychotic [94].

Conjugated estrogen 0.625–1.875 mg was used successfully, without any specific side effects, in two 78-year-old patients respectively with Alzheimer’s disease and vascular dementia who had failed to respond to various antipsychotics [95].

##### Gonadotropin-releasing hormone analogs

Gonadotropin-releasing hormone analogs (GnRHA) suppress testosterone production by stimulating the secretion of pituitary LH and FSH, with subsequent increase in estrogen levels and decrease of testosterone.

There are two case reports of the use of leuprolide acetate in ISB: the first describes improvement after leuprolide acetate 7.5 mg intramuscularly every month in a 43-year-old patient with dementia and Klüver-Bucy syndrome who had not responded to pindolol [96]; the second is in a 73-year-old man with vascular dementia and comorbid depression who did not respond to MPA, but in whom leuprolide at the same dose, 8 weekly was beneficial [23].

Safety and side effects are important issues in prescribing hormonal agents. In females, there may be menopausal symptoms, though this may be less common in post-menopausal women. Osteoporosis, edema, weight change, and mood changes may occur. Costs of GnRHA can be high, although estrogens and antiandrogens are generally less expensive.

### Beta-blockers

Beta-blockers can cause sexual dysfunction and reduced sexual behavior, possibly via a decrease of the adrenergic drive [97].

Propranolol 360 mg was effective in controlling verbal and physical aggression, but not sexually aggressive and inappropriate behavior, which only responded to treatment with leuprolide [96].

In a 75-year-old demented man with aggressive and hypersexual behaviors which had not responded to haloperidol and hydroxyzine, both agitation and hypersexual behavior diminished within 2 weeks of adding pindolol 40 mg to his drug regime [98].

Beta-blockers slow the heart rate and are contraindicated in patients with heart block and asthma. They can cause coldness, fatigue, and sleep disturbance. Most are inexpensive agents.

### Combination therapy

Combination therapy with various medications described above has been suggested for persistent ISB [42]. Several case reports describe combinations of drugs to treat these symptoms [99], but clinicians should be cautious with polypharmacy in the elderly, especially when it involves carbamazepine or cimetidine, as these drugs have important effects on hepatic metabolism and pharmacological interactions [42], and elderly people are particularly vulnerable to adverse effects of medication.

## Discussion

Case reports and case series describe successful treatment of ISB with a range of classes of medication, including antidepressants, antipsychotics, anticonvulsants, cholinesterase inhibitors, hormonal agents, and beta-blockers. All of these agents can have adverse effects, and none of them emerges as a clear first choice for pharmacological management of ISB.

The quality of evidence in this area is limited to case reports and case series. Studies are further limited by a lack of a widely agreed definition of ISB, and only very limited objective methods for measuring it. There are no randomized controlled trials. The present paper reviews published data on pharmacological interventions for ISB, but is not intended to provide a complete review of a field where it is essential to ensure that there is a full assessment of the problem, careful attention to ethics and consent, and careful monitoring of response.

As there is little evidence to suggest that one type of agent is more efficacious than another in this area, it is important to pay particular attention to risks in making a choice of agent. There will need to be a careful balancing of risk and potential benefit. Although there is a lack of published evidence to show that behavioral, supportive, and educational approaches to managing ISB are effective, clinicians may wish to start by considering the place of non-drug management. Involvement of staff, families, and caregivers in discussion will be important. It is important to rule out treatable causes of ISB such as pain, boredom, lack of stimulation, and lack of an outlet for affectional needs. Only then should medication be considered. Although all agents carry risks, the overall risks associated with SSRI antidepressants are relatively lower than with other drugs, and these may be a reasonable first choice. Cholinesterase inhibitors may be a sensible next step. Antipsychotics and hormonal agents may have very significant side effects and should be used with caution. In all cases, it is helpful to define target symptoms or behaviors and to provide care staff or caregivers with a simple means of recording these so that the success or otherwise of treatment can be evaluated. Attention must be paid to the emergence of side effects. Most of the patients involved are likely to be old and often frail so that it is wise to start prescribing at low dose and to increase only very slowly.

There is a need for a better understanding of the nature of ISB and better methods of describing and measuring it. Only then will it be possible to carry out well-designed, double-blind randomized controlled trials in order to evaluate the effectiveness, safety, and tolerability of the treatments of ISB.
